# Nursing intervention for severity of urinary incontinence

**DOI:** 10.6026/973206300221014

**Published:** 2026-02-28

**Authors:** S Malarvizhi, Anuratha Matipol Dharmalingam

**Affiliations:** 1 Department of Medical Surgical Nursing, PSG College of Nursing, Coimbatore, Affiliated to The Tamil Nadu Dr. MGR Medical University, Chennai, India

**Keywords:** Urinary incontinence, severity of urinary incontinence, postmenopausal urinary incontinence, nursing intervention

## Abstract

Urinary incontinence (UI) is a highly prevalent health problem among postmenopausal women ending in silent suffering. Therefore, it is of interest to assess the feasibility and effect of tailored, Evidence-based nursing intervention on severity of UI symptoms. Quasi experimental time series repeated measures design was used. Two old age homes were randomly assigned for an interventional and control setting. Ten post-menopausal women, selected by non-randomized purposive sampling from each setting were assigned to an interventional (n=10) and control group (n=10) respectively. The severity of Urinary incontinence in both groups was assessed using International Consultation on Incontinence (ICIQ -UI SF) questionnaire. The interventional group women received the nursing intervention for 12 weeks, whereas the control group continued to receive the usual care. The severity of urinary Incontinence was assessed at the end of 4th, 8th and 12th weeks. Repeated measures ANOVA of pre-test and post-test-III scores in Interventional group (F= 18.46, P ≤ 0.001) was significant. In control group, there was no significant difference (F = 1.47, P ≥ 0.05).

## Background:

Menopause is as an inevitable part of biological aging, ceasing the reproductive functioning in a woman. During this phase, rapid physiological changes occur, as a result of low oestrogen levels causing atrophic changes and muscle weakness [1]. Urinary incontinence (UI) is defined by the International Continence Society (ICS) as involuntary leakage of urine. The three subtypes of UI are stress urinary incontinence, urge urinary incontinence and mixed urinary incontinence [2]. Urinary Incontinence (UI), affects at least 50% of the women globally. Still, the societal stigma associated with the discussion of the symptoms results in the reluctance of numerous women to seek professional medical care [3]. Especially among the Indian women, the treatment seeking behaviour is considerably low, in spite of the high prevalence rates. The factors attributing to this fact are non-availability of female doctors in the periphery, shyness, anxiety about medical expenses and considering UI as part of normal aging [4]. UI is commonly treated by conservative methods, drugs or surgical management. Conservative management is the first line management recommended for women with UI. Strong evidences suggest that management of UI is highly effective if provided with the support of health team members incorporating measures to encourage consistent adherence [5]. Therefore, it is of interest to assess the impact of a well-tailored, comprehensive, holistic nursing intervention to support the postmenopausal women in overcoming urinary incontinence in their own place of residence.

## Methodology:

## Research design:

A Quasi experimental - time series with repeated measures design was used.

## Setting:

The study was conducted in two selected old age homes which were randomly assigned to experimental and control setting.

## Participants and sampling:

The pilot study was conducted among twenty post-menopausal women with urinary incontinence selected using non randomized purposive sampling technique. Ten post-menopausal women who met the inclusion criteria from the control setting were selected for the control group (n=10). Ten women who met the criteria from the Interventional setting were selected for the Interventional group (n=10).

## Research instrument:

The severity of urinary incontinence was assessed using International Consultation on Incontinence (ICIQ -UI SF) short form questionnaire in both groups. The severity of Urinary Incontinence was classified into four groups; slight, moderate, severe and very severe.

## Intervention:

Pretest for both groups was done using the ICIQ -UI -SF Questionnaire the women in the Interventional group received the Nursing Intervention for 12 weeks under the guidance and supervision of the nurse investigator. The control group received the routine care given by the old age home care takers. Post-tests I, II and III were conducted at the end of every four weeks for 3 consecutive months using the same questionnaire.

## Ethical considerations:

Ethical approval was obtained from IHEC. Permission of the old age home authorities and Informed consent from the participants were obtained after clear explanation.

## Data analysis:

Statistical analysis was done using descriptive statistics for demographic data and inferential statistics for analysis of urinary incontinence among the two groups. Independent t-test was used for between group comparisons and one way repeated measure ANOVA F test was used to measure the comparison of mean symptom severity scores from pretest to post test in both groups.

## Results and Discussion:

This pilot study examined the impact of nursing intervention on the severity of Urinary Incontinence among post-menopausal women residing in old age homes. About half of the women in Interventional group 5(50%) and majority of the women in control group 8(80%) were aged between 50-60 years. Majority of the women (interventional group-70% and control group-80%) had primary education and only few were graduates. None of the women had spouses in both groups. Few women in both groups 3(30%) were living in the old age homes for more than 10 years. Two women (20%) in the interventional group were equally obese and overweight whereas only 1(10%) woman was overweight and none were obese in control group. More than half of the women in the interventional group 6(60%) and 7(70%) in control group had history of vaginal delivery. Most of the women in both groups (interventional group -70%, control group-90%), had no history of abdominal or pelvic surgeries. Almost all women 9(90%) in both groups had no habit of performing any form of physical exercise. Majority of the women in the interventional group 7(70%) and 9((90%) women in control group had not shared about their incontinence to anyone prior. This result is aligned with the recent evidences which states that most of the women with urinary incontinence do not disclose symptoms or seek professional help. In a recent study, Fakri et al. (2023) stated that shame and embarrassment were the reasons for not sharing the issue in spite of the persistence of symptoms [6]. Table 1 describes the comparison of interventional and control group symptom severity score among post-menopausal women with Urinary Incontinence during pretest, post-test-I, post-test-II and post-test-III using student independent t test.

In the pretest, interventional group samples had mean symptom severity score of 9.20 + 1.75 and control group had 8.80 + 1.75 with a mean difference of 0.40. There was no statistically significant difference (t=0.51 at P > 0.05). In the post-test I and post-test II, the mean differences were 0.90 (t=1.17) and 1.20 (t=1.94) at P>0.05, which were also statistically non-significant. In the post-test- III, the mean difference of symptom severity score among both groups was 2.00. This difference is large and is statistically significant (t=2.25 at P≤ 0.05) which highlights that the Nursing Intervention was effective in reducing symptom severity among postmenopausal women with urinary incontinence when performed consistently. Table 2 shows the analysis of the mean overall symptom severity scores by repeated measures F-test. In the Interventional group there was a statistically significant difference between pre-test and post-test-III (F= 18.46, P>0.05) scores. In control group, there was no significant difference between pre-test and post-test-III (F= 1.47, P ≥ 0.05) scores indicating without intervention there were no changes. The repeated measures analysis confirms that the interventional group had a progressive decline in the severity of symptoms while the scores of the control group revealed that there was no significant change. The interventional group exhibited a significant improvement with a mean reduction of 30.43% in post severity scores whereas there was only a negligible difference in control group (4.5%). This result is supported by an RCT conducted by Gonzaga [7] which compared the pelvic floor exercises with Pilates and demonstrated a significant reduction in urinary incontinence after a 12-week trial in both groups revealing, exercise-based interventions have clinically meaningful benefits compared to baseline. This finding also aligns with the result of a pilot randomized control trial conducted by Li *et al.* in Taiwan [8] which showed an 8-week multimodal programme reduced severity of symptoms of UI. The result also concurs with systematic review conducted by Brown *et al.* reporting that the intensity, frequency, individual supervision of Pelvic floor muscle training combined with behavioural modification is effective in decreasing the severity of UI [9]. The observation strongly supports the views of Alrashdi *et al.* [10] which state that by providing humanistic and comprehensive nursing care, women can handle the difficulties of urinary incontinence with confidence enhancing positive health outcomes.

## Conclusion:

The severity of urinary incontinence in post-menopausal women can be alleviated by supportive interventions, tailored to meet individual needs when incorporated consistently. Considering the increased prevalence yet underreported nature of urinary incontinence in postmenopausal women, the result of this study highlights the significant role of nurses in identifying and planning tailored interventions to meet the needs of less privileged women. In spite of the limitations of a smaller sample size, this study establishes a concrete foundation for larger scale research.

## Figures and Tables

**Table 1:** Comparison of Interventional and Control group mean Symptom Severity (ICIQ-UI-SF) score of Pretest, Post-test-I, Post-Test II and Post-test-III. n=20

**Symptom severity**	**Group**				**Mean Difference**	**Student independent t-test**
	**Interventional (n=10)**		**Control (n=10)**			
	**Mean**	**SD**	**Mean**	**SD**		
Pretest	9.2	1.75	8.8	1.75	0.4	t=0.51 P=0.62 DF=18 (NS)
Post-test-I	7.8	1.75	8.7	1.7	-0.9	t=1.17 P=0.25 DF=18 (NS)
Post-test-II	7.3	1.42	8.5	1.35	-1.2	t=1.94P=0.06 DF=18 (NS)
Post-test-III	6.4	1.43	8.4	2.41	-2	t=2.25 P=0.05* DF=18 (S)
NS = Not significant;
S= significant P>0.05

**Table 2:** Comparison of mean symptom severity (ICIQ-UI-SF) score of experimental and control group in pretest, post-test-I, post- test-II And post-test-II n=20

**Group**	**Pre-test**		**Post-test-I**		**Post-test-II**		**Post-test-III**		**MD**	**One-way Repeated measures ANOVA**
	**Mean**	**SD**	**Mean**	**SD**	**Mean**	**SD**	**Mean**	**SD**		**F-test**
**Interventional**	**9.2**	**1.75**	**7.8**	**1.75**	**7.3**	**1.42**	**6.4**	**1.43**	**2.8**	**F=18.46**
										p=0.001*** (S)
Control	8.8	1.75	8.7	1.7	8.5	1.35	8.4	2.41	0.4	F=1.47
										p=0.22
										(NS)
MD-Mean difference;
NS = Not significant;
S= significant P>0.05
